# Expanding the genetic landscape of inherited metabolic diseases using long-read sequencing and transcriptomic profiling

**DOI:** 10.1038/s41431-025-01995-7

**Published:** 2026-01-26

**Authors:** Alejandro Soriano-Sexto, Obdulia Sánchez-Lijarcio, Leonardo Beccari, Natalia Castejón-Fernández, Fátima Leal, Patricia Alcaide, Belén de la Morena-Barrio, María del Pilar Bahíllo-Curieses, Patricia Correcher, Rafael Hencke-Tresbach, Laura López, Elena Martín-Hernández, Raquel Yahyaoui, Magdalena Ugarte, Pilar Rodríguez-Pombo, Belén Pérez

**Affiliations:** 1 Centro de Diagnóstico de Enfermedades Moleculares, Centro de Biología Molecular, Universidad Autónoma de Madrid, CIBERER, IdiPAZ, Madrid, Spain; 2 Centro de Biología Molecular, Consejo Superior de Investigaciones Científicas, Madrid, Spain; 3 Servicio de Hematología y Oncología Médica, Hospital Universitario Morales Meseguer, Centro Regional de Hemodonación, Universidad de Murcia, IMIB-Arrixaca, CIBERER, Murcia, Spain; 4 Servicio de Pediatría, Endocrinología Pediátrica, Hospital Clínico Universitario, Valladolid, Spain; 5 Laboratorio de Metabolopatías, Hospital Universitario La Fe, Valencia, Spain; 6 Sección de Neurología Pediátrica, Hospital Infantil Universitario Niño Jesús, Madrid, Spain; 7 Sección de Enfermedades Mitocondriales-Metabólicas Hereditarias. Instituto de investigación imas12. Hospital Universitario 12 de Octubre, Madrid, Spain; 8 Departamento de Biomedicina y Odontología, Facultad de Ciencias Biomédicas y Deporte. Universidad Europea de Andalucía, Laboratorio de Metabolopatías. Hospital Regional Universitario de Málaga. Instituto de Investigación Biomédica de Málaga (IBIMA-Plataforma BIONAND), Málaga, Spain

**Keywords:** Metabolic disorders, Clinical genetics, Mutation

## Abstract

Although next-generation sequencing has emerged as a powerful tool for diagnosing rare diseases (RD), many cases of inherited metabolic diseases (IMD) remain unsolved, hindering the diagnosis, clinical and therapeutic management of the patients. The primary aim of this study is to address the most elusive cases by applying long-read sequencing (LRS) targeted to the gene of interest on seven patients (*FARS2*, *GYS2*, *PEX1*, *SLC2A1*, *AGL*, *ACAT1*, and *ACADM*), identifying six novel pathogenic variants including two intronic variants, a structural variant and three transposable elements (TE) insertions. In addition, we have demonstrated the effect on splicing of an exonic variant previously reported as missense. Functional genetic tests specific for the expected effect of each variant of uncertain significance were designed, such as minigenes analysis or chromatin conformation capture assay. From the TE insertions, two were located in the genomic region of *GYS2* or *PEX1*, causing a reduction in their mRNA expression. The third was located 7.6 kb downstream of *SLC2A1*; it alters the interaction between the *SLC2A1* promoter and its distal regulatory element via the establishment of a loop with the 3’ border of the native topologically associating domain. This study shows that the combination of LRS and functional genetic assays confers a powerful approach for expanding the mutational spectrum of IMD, adding data to improve the diagnosis of this large group of RD.

## Introduction

Inherited metabolic diseases (IMD) are a diverse group of nearly 2000 disorders that collectively represent somewhat common disorders [1]. Currently, the gold standard for diagnosis involves identifying abnormal levels of biochemical markers in newborn screening or after the first manifestation of the symptoms. This diagnosis should be confirmed by molecular-genetics technologies [2].

The emergence of next-generation sequencing (NGS) has become the gold standard for genetic testing. Exome sequencing (ES) is the most commonly used genetic test in the clinical setting; however, it leaves a significant number of cases undiagnosed [3]. Thus, other technologies that target a wider spectrum of genetic variation are needed [3].

The variants that fall in non-coding regions can impact various mechanisms, such as splicing [4] or transcription regulation. In complex eukaryotes, gene expression is regulated by non-coding sequences known as *cis*-regulatory elements (CRE), including promoters and enhancers [5]. While the basal promoters are located in proximity to the gene’s transcription start site (TSS), enhancers are often found in distant regions, requiring the 3D conformation of chromatin to bring them into physical proximity. Topologically Associating Domains (TADs) delimit, in large part, gene’s regulatory landscapes and are established by the activity of CCCTC binding factor (CTCF) and cohesin [6]. Thus, variants affecting CRE activity or TAD organization may contribute to disease [7].

Short-read genome sequencing (srGS) increases the diagnostic rate compared to exome sequencing, some cases remain incomplete [8]. One of the main drawbacks of srGS is its limited read length [9] that prevents the detection of some structural variants (SV) and tandem repeat expansions (TREs). Long-read sequencing (LRS) enables mapping of repetitive or duplicated regions, detection of TREs and SV at the same time, resolving break-points at nucleotide resolution [10]. Additionally, the sequencing of native molecules eliminates PCR bias, conferring this technology the ability to analyze epigenetic mechanisms [10] and allows phasing of all types of variants detected.

SVs are defined as differences between huge DNA segments, normally bigger than 50 bp, across genomes. They are usually produced by errors during DNA replication or repair. Genomic regions with a high percentage of homology can lead to erroneous recombination events, resulting in different types of SV. Transposable elements (TE) are sequences with a high number of repeats that comprise around two-thirds of the human genome and can serve as homology regions for the generation of SV [11–14].

Among the different classes of TE, only non-Long-Terminal Repeats retrotransposons, such as Long Interspersed Nuclear Elements (LINEs), specifically L1 subfamily, Alu, and SINE-VNTR-Alu (SVA), maintain the ability to move in the human genome [15, 16]. The insertion of TE fragments in the genome can lead to disease by exon interruption, alteration of splicing, epigenetic changes, deletion production or even by changing the chromatin conformation [11, 12, 16, 17].

New technologies have significantly increased the number of identified variants, which require the determination of their effect. While in silico predictors may help prioritize them, they are not sufficient to establish their pathogenicity. Therefore, functional genetic tests are necessary [18], such as minigenes for splicing variants, reporter assays for changes in promoters or enhancers, chromatin conformation capture techniques to analyze alterations in chromatin 3D interactions, among others [19].

In this work, taking advantage of the metabolic profiling of our participants, we have been able to focus our study on specific *loci*, which removes a limitation to clinical use of LRS. We have tested the potential use of LRS targeted to specific *loci* in combination of a comprehensive set of functional and metabolomics assays to reduce the diagnosis gap in IMD.

## Materials and methods

### Participants

Participants’ fibroblasts (from all cases except for P2, from which a hepatic biopsy was used) were obtained from skin biopsies. Cultures were maintained in Minimal Essential Medium (MEM) supplemented with 10% fetal calf serum, 1% glutamine, 100,000 U/L penicillin and 100 mg/dL streptomycin. Cells were maintained in a humidified incubator held at 5% CO_2_ and 37 °C.

### RNA studies

RNA was extracted using the RNeasy Micro Kit (Qiagen, Hilden, Germany) according to the manufacturer’s instructions. A total of 1.5 μg of RNA were used for cDNA retrotranscription using the SuperScript VILO cDNA Synthesis Kit (Thermo Fisher Scientific, Waltham, MS, USA) following the manufacturer’s protocol. Fragments of interest were amplified by PCR using FastStart Taq DNA Polymerase (Roche Applied Science, Indianapolis, IN, USA) and specific primers and Sanger sequenced using the BigDye Terminator Cycle Sequencing Kit (Applied Biosystems, Foster City, CA, USA).

Differential gene expression was analyzed by RT-qPCR. This assay was performed starting with 250 ng total RNA that was transcribed to single-stranded cDNA using NZY First-Strand cDNA Synthesis Kit (NZYTech, Lisbon, Portugal) following the manufacturer’s instructions. Specific primers were designed for *AGL* (HGNC:321) and *SLC2A1* (HGNC:11005)*. GUSB* (HGNC:4696) was used as an endogenous control. qPCR experiments were performed in a LightCycler® 480 Instrument (Roche Applied Science) using PerfeCTa SYBR® Green FastMix (Quantabio, Beverly, MA, USA), following the LightCycler® manufacturer’s instructions except for the amplification step which was modified to 10 s at 95 °C, 30 s at 60 °C, and 30 s at 72 °C. Cycle threshold values were obtained and analyzed using the 2^−ΔΔCt^ method. Primers used for amplification will be sent upon request.

### Long-read sequencing

High-purity DNA was extracted from peripheral blood or from participant-derived fibroblasts using the MagNA Pure Compact System and either the MagNA Pure Compact Nucleic Acid Isolation Kit I-Large Volume or the MagNA Pure Compact Nucleic Acid Isolation Kit I (Roche Applied Science) following the manufacturer’s instructions.

Libraries were prepared with 1d Ligation Library Prep Kit (Oxford Nanopore Technologies [ONT], Oxford, UK) utilizing LSK114 for P5 and P7 and LSK109 for the rest, and were sequenced in a MinION or PromethION P2 device (ONT) using R9.4.1 for P1, P2, P3, P4 and P6 and R10.4.1 for P5 and P7. For sequencing and enrichment of the target region, the adaptive sampling tool [20] was used, implemented in the MinKNOW software (ONT), using a bed file with the genomic coordinates of interest. Bioinformatic analysis of the generated data was performed with a pipeline from Longseq Applications that consisted of: i) base calling using Dorado base caller, which is integrated within the MinKNOW software [21] using FAST basecaller for P1, P2, P3, P4 and P6 and HAC for P5 and P7; ii) alignment to the human reference assembly (GRCh38) using Minimap2 [22]; iii) variants calling with Sniffles2 [23] software for SVs, iv) Clair3 was used for SNV calling and phasing of alignments [24], v) annotation of the variants was done using SnpEff, SnpSift, VEP. For visual inspection and interpretation of long-read alignments, Integrated Genome Viewer (IGV) was used [25]. The data quality was assessed using MinION QC and QualiMap tools. Methylation calls were only obtained for P7.

All variants were named following the Human Genome Variation Society (HGVS) recommendations and verified using the software VariantValidator [26].

### Minigene studies

To examine the splicing pattern in vitro, the pSPL3 vector was used (Exon Trapping System, Gibco, BRL, Carlsbad, CA, USA). The fragment containing *ACAT1* (HGNC:93) exon 10 and adjacent intronic regions was isolated from the case and cloned into the pGEMT-Easy vector (Promega, Madison, WA, USA) and the alleles isolated. The insert was excised with *Eco*RI (Roche Applied Science), purified using the QIAquick Gel Extraction Kit (Qiagen), and subsequently cloned into the pSPL3 vector dephosphorylated with Thermosensitive Alkaline Phosphatase (Promega). Ligation was performed using the Rapid DNA Ligation Kit (Thermo Fisher Scientific). Restriction enzyme analysis and Sanger sequencing were used to select the clones containing the desired wild-type and mutant alleles. Two µg of the wild-type or mutant minigene were then transfected into the HepG2 cell line using JetPEI transfection reagent (Polyplus-Transfection, Illkirch, France) following the manufacturer’s protocol. Cells were harvested 48 h post-transfection. Transcription profile studies were performed as described in the section RNA studies, and amplification was performed with vector internal primers.

### Luciferase reporter assay system

The promoter sequence, including the potential TSS of *ACADM*, was identified using the Eukaryotic Promoter Database (EPD) (https://epd.epfl.ch//index.php) and the ENCODE Candidate *Cis*-Regulatory Elements (cCRE) registry on the University of California, Santa Cruz genome browser (https://genome.ucsc.edu/).

The selected region was amplified both from healthy control and patient fibroblasts using specific primers carrying the Gateway attB1 and attB2 sites and cloned into the pDONR^TM^221 vector (Thermo Fisher Scientific) using Gateway™ BP Clonase™ II (Thermo Fisher Scientific) following the manufacturer’s recommendations. The obtained vector was transformed in the DH5α strain. The NC_000001.11(NM_000016.5):c.-440T>C variant was both introduced in the control DNA using QuikChange Lightning Site-Directed Mutagenesis Kit (Agilent Technologies, Santa Clara, CA, USA) and isolated from the case. Next, the insert was moved to the pIRIGF vector (Addgene, Watertown, MA, USA) by recombination using Gateway™ LR Clonase™ II (Thermo Fisher Scientific) following the manufacturer’s instructions. Clones were confirmed by Sanger sequencing.

The HepG2 cell line was then transfected with 2 µg of wild-type or mutant constructs using JetPEI transfection reagent (Polyplus-Transfection) following the manufacturer’s indications. Cells were harvested 48 h post-transfection.

Firefly and *Renilla reniformis* luciferase activities were assessed using the Dual-Luciferase Reporter Assay System (Promega) following the manufacturer’s indications, and detected using FLUOstar OPTIMA microplate reader (BMG Labtech, Durham, NC, USA).

### Circular chromatin conformation capture

Circular Chromatin Conformation Capture coupled to NGS (4Cseq) experiments were performed and analyzed as in our previous study [27]. Viewpoint-specific primers for the *SLC2A1* promoter or CRE region are indicated in Supplemental Information (Table 2)

### AI and AI-assisted technologies in the writing process

Grammarly has been used to improve the readability of the manuscript. After using this tool, the authors reviewed and edited the content as needed and take full responsibility for the content of the publication.

## Results

In this study, we included seven participants (P) (Table 1) presenting clinical and/or biochemical suspicion of an IMD. The possible diagnoses were a combined oxidative phosphorylation deficiency (MIM#614946), glycogen storage disease (MIM#240600/MIM#232400), peroxisome biogenesis disorder (MIM#214100), glucose transporter 1 deficiency syndrome (GLUT1-DS; MIM#606777), alpha-methylacetoacetic aciduria (MIM#203750), or medium-chain Acyl-CoA dehydrogenase deficiency (MIM#201450). All these diseases are associated with an autosomal recessive inheritance pattern, except for GLUT1-DS, which has an autosomal dominant inheritance.

**Table 1:** Participants (P) analyzed in this study with their biochemical and clinical data, biochemical suspicion and the age at diagnosis.

Participant	Biochemical analysis	Clinical data	Biochemical suspicion	Age at biochemical diagnosis	Age at genetic diagnosis
P1	Citric acid: 346 mmol/mol creat. Fumaric acid: 83 mmol/mol creat. 2-Oxoglutaric acid: 209 mmol/mol creat. Blood pyruvate: 0.114 mM Cerebrospinal fluid pyruvate: 0.186 mM Blood lactate: 4.88 mM Cerebrospinal fluid lactate: 3.93 mM β-hydroxybutyrate: 0.529 mM Acetoacetate: 0.137 mM	Metabolic acidosis HP:0001942 Hyponatremia HP:0002902 Global developmental delay HP:0001263 Generalized hypotonia HP:0001290 Seizures HP:0001250 Abnormality of extrapyramidal motor function HP:0002071 EEG with abnormally slow frequencies HP:0011203 Intraventricular haemorrhage HP:0030746 Abnormal delivery HP:0001787 (foetal suffering)	Congenital lactic acidosis	1 year	27 years
P2	Glucose: 37 mg/dL	Ketotic hypoglycemia HP:0012734 Postprandial hyperglycemia HP:0011998 Short stature HP:0004322	Glycogen storage disease	4 years and 11 months	13 years
P3	Hexacosanoic acid (C26:0): 5.640 µmol/L C24/C22 ratio: 1.274 C26/C22 ratio: 0.297	Global developmental delay HP:0001263 Hypotonia HP:0001252 Elevated circulating hepatic transaminase concentration HP:0002910 Hepatomegaly HP:0002240 Splenomegaly HP:0001744 Failure to thrive HP:0001508 Polymicrogyria HP:0002126 Decreased liver function HP:0001410 Brain imaging abnormality HP:0410263 Abnormal seventh cranial physiology HP:0010827 Increased circulating very long-chain fatty acid concentration HP:0033643	Peroxisome biogenesis disorder	6 months	3 years
P4	Cerebrospinal fluid glucose: 45 mg/dL Cerebrospinal fluid to serum blood glucose ratio: 0.53 Cerebrospinal fluid lactate: 11.0 mg/dL	Microcephaly HP:0000252 Epileptic encephalopathy HP:0200134 Generalized myoclonic seizure HP:0002123 Atypical absence seizure HP:0007270 Delayed speech and language development HP:0000750 EEG abnormality HP:0002353 Short attention span HP:0000736 Motor stereotypy HP:0000733 Recurrent hand flapping HP:0100023 Hypoglycorrhachia HP:0011972	Glucose transporter 1 deficiency syndrome	2 years	12 years
P5	Fasting hypoglycemia (HP:0003162) with ketonuria (HP:0002919) Serine: 266.5 µmol/L Glycine: 628,1 µmol/L Alanine: 940.4 µmol/L Blood methionine: 43.1 µmol/L Urine methionine: 67.3 mmol/mol creat. Isoleucine: 17.8 mmol/mol creat. Ornithine: 19.0 mmol/mol creat. Lactic acid: 227 mmol/mol creat. Hexanoyl glycine: 2 mmol/mol creat.	Hepatomegaly HP:0002240 Fasting hypoglycemia HP:0003162 with Ketonuria HP:0002919 Elevated circulating hepatic transaminase concentration HP:0002910 Elevated circulating creatine kinase concentration HP:0003236 Ventricular septal hypertrophy HP:0005144	Glycogen storage disease	5 months	19 years
P6	3-OH-butyric acid: 24847 mmol/mol creat. Acetoacetic acid: 2408 mmol/mol creat. Lactic acid: 2463 mmol/mol creat. 2-OH-butyric acid: 74 mmol/mol creat. 2-Me-3-OH-butyric acid: 255 mmol/mol creat. 3-OH-isovaleric acid:484 mmol/mol creat. Ethylmalonic acid: 33 mmol/mol creat. Adipic acid: 110 mmol/mol creat. 2-OH-adipic acid: 25 mmol/mol creat. Detection of 2-Me-acetoacetic acid and 3-oxovaleric acid Reduced ACAT1 enzymatic activity	Acidosis HP:0001941 Ketosis HP:0001946 Tachypnea HP:0002789 Respiratory alkalosis HP:0001950	Alpha-methylacetoacetic aciduria	2 years	6 years
P7	C6 hexanoyl carnitine: 0.17 µmol/L C8 octanoyl carnitine: 1.06 µmol/L C10 decanoyl carnitine: 0.76 µmol/L 7%ACADM enzymatic activity	Seizure HP:0001250 Myoclonus HP:0001336 Short attention span HP:0000736 Microcephaly HP:0000252 Abnormal cerebral white matter morphology HP:0002500 Periventricular white matter hyperintensities HP:0030891 Exercise intolerance HP:0003546 Elevated circulating medium-chain acylcarnitine concentration HP:0035017	Medium-chain acyl-CoA dehydrogenase deficiency	6 years	20 years

Normal values: citric acid (<300 mmol/mol creat.), fumaric acid (<10 mmol/mol creat.), 2-Oxoglutaric acid (<150 mmol/mol creat.), blood pyruvate (0.08 ± 0.03 mM), cerebrospinal fluid pyruvate (0.077 ± 0.029 mM), blood lactate (1.08 ± 0.5 mM), cerebrospinal fluid lactate (1.63 ± 0.39 mM), β-hydroxybutyrate (<0.2 mM), acetoacetate (<0.1 mM), glucose (110–200 mg/dL), hexacosanoic acid (C26:0) (<0.980 µmol/L), C24/C22 ratio (<0.990), C26/C22 ratio (<0.021), cerebrospinal fluid glucose (62.2 ± 17.1 mg/dL), cerebrospinal fluid to serum blood glucose ratio (0.585 ± 0.155), cerebrospinal fluid lactate (16.2 ± 4.5 mg/dL), serine (115.0 ± 33.1 µmol/L), glycine (200.2 ± 71.5 µmol/L), alanine (262.3 ± 98.5 µmol/L), blood methionine (18.9 ± 6.3 µmol/L), urine methionine (12.9 ± 8.9 mmol/mol creat.), isoleucine (5.4 ± 3.5 mmol/mol creat.), ornithine (6.2 ± 5.4 mmol/mol creat.), lactic acid (1–113 mmol/mol creat.), hexanoyl glycine (not detectable), 3-OH-butyric acid (0–17 mmol/mol creat.), acetoacetic acid (0–7 mmol/mol creat.), 2-OH-butyric acid (0–4 mmol/mol creat.), 2-Me-3-OH-butyric acid (1–29 mmol/mol creat.), 3-OH-isovaleric acid (3–40 mmol/mol creat.), ethylmalonic acid (0–18 mmol/mol creat.), adipic acid (1–47 mmol/mol creat.), 2-OH-adipic acid (not detectable), 2-Me-acetoacetic acid (not detectable), 3-oxovaleric acid (not detectable), C6 hexanoyl carnitine (0.00–0.13 µmol/L), C8 octanoyl carnitine (0.01–0.25 µmol/L), C10 decanoyl carnitine (0.04–0.41 µmol/L).

*ACAT1* Acetyl-CoA Acetyltransferase 1, *ACADM* Acyl-CoA Dehydrogenase Medium Chain.

Following the clinical and/or biochemical diagnosis, ES analysis identified a heterozygote pathogenic variant in six participants (five exonic and one intronic), which are associated with an autosomal recessive inheritance, while no pathogenic variants were found in the patient with GLUT1-DS possible diagnosis (Table 2). All variants had been previously described in HGMD (2025 v3), except for *ACAT1* NM_000019.4:c.841G>A. Despite this clinical testing, all cases remained unsolved.

**Table 2:** Participants (P) analyzed in this study with the results from exome sequencing (ES), RNA analysis, targeted long-read sequencing (T-LRS) and the validated effect from the variants detected by T-LRS.

Participant	Gene	ES	RNA analysis	T-LRS	Variant effect
P1	*FARS2*	c.737C>T;c.1082C>T p.(Thr246Met); p.(Pro361Leu)	r.49_904del	c.905-5741_1065+1116dup	Exon 5 duplication in cDNA: r.(905_1065dup)
P2	*GYS2*	c.1436C>A p.(Pro479Gln)	r.1230_1308del	c.1300_1301ins[PP887427.1:g.1_1518]	Skipping of exon 10: r.1230_1308del
P3	*PEX1*	c.2097dup p.(Ile700Tyrfs*42)	ASE	SVA insertion in intron 8	Possible pseudoexon insertion
P4	*SLC2A1*	–	20% *SLC2A1* expression	LINE insertion in 3’ region	Alteration of chromatin 3D structure
P5	*AGL*	c.4260-1G>T p.?	r.4260_4347del 4% *AGL* expression	c.3259+927A>G	Pseudoexon insertion: r.3259_3260ins[3259 + 818_3259 + 922]
P6	*ACAT1*	c.841G>A p.(Ala281Thr)	ASE	c.941-60T>C	Skipping of exon 10: r.941_1005del
P7	*ACADM*	c.542A>G p.(Asp181Gly)	r.542_599del^a^ r.542_628del^a^ ASE	c.-440T>C/c.945+803A>C	No clear function detected

*ASE* allele-specific expression, *SVA* SINE-VNTR-Alu, *LINE* Long Interspersed Nuclear Element*, FARS2* NC_000006.12(NM_006567.5), *GYS2* NC_000012.12(NM_021957.4), *PEX1* NC_000007.14(NM_000466.3), *SLC2A1* NC_000001.11 (NM_006516.4), *AGL* NC_000001.11(NM_000642.3), *ACAT1* NC_000011.10(NM_000019.4), *ACADM* NC_000001.11(NM_000016.5).

^a^Effect detected in this work.

### Transcriptional studies

In an attempt to identify the cause of the disease in the seven cases, we conducted RNA studies in participant-derived fibroblasts to evaluate the effect of possible variants affecting expression, splicing, or mRNA stability. For P1, the transcriptional profile was previously reported [28], detecting two amplicons: one containing the two single-nucleotide variants (SNVs) detected in ES and a smaller amplicon which exhibited a skipping of exons 3 and 4 and a portion of exon 2 (r.49_904del) of *FARS2* (HGNC:21062).

**Fig. 1:**
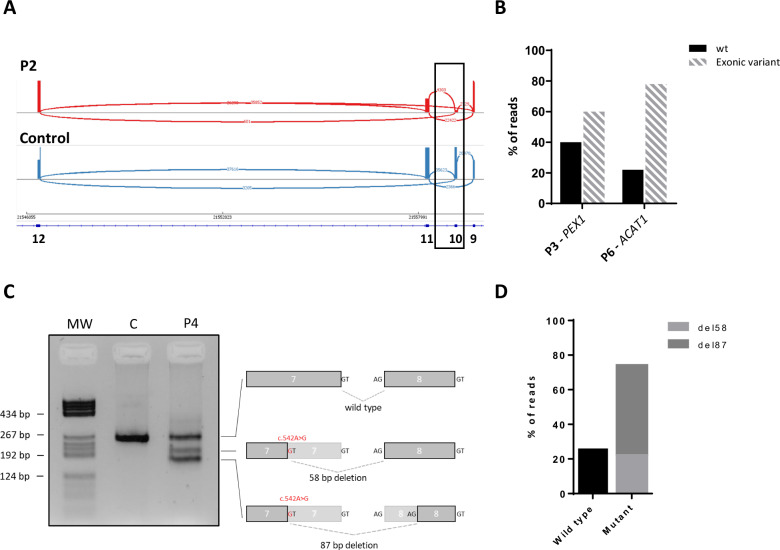
Transcriptional analysis of participants (P) 2, 3, 4 and 6. **A** Sashimi plot obtained for *GYS2* in P2. Exon 10 skipping is marked with the black rectangle. **B** Quantification of the percentage of reads of cDNA of *PEX1* and *ACAT1* with or without (wt) the exonic variants detected in P3 and P6. **C** Transcriptional profile of the *ACADM* gene performed in RNA isolated from control (C) or P7 fibroblasts. Wild-type or aberrant transcripts of *ACADM* are represented on the right with the exonic variant NM_000016.5:c.542A>G marked in red. Deleted regions are depicted in light gray. All bands have been Sanger sequenced. **D** Quantification of the percentage of reads obtained after next-generation sequencing of the *ACADM* cDNA obtained from case P7. The percentage of reads with a normal splicing pattern or mutant with either the 58 bp or the 87 bp deletions are represented.

We detected exon 10 skipping in *GYS2* (HGNC:4707) (Fig. 1A), and no variants in exon 10 and flanking regions were detected in *GYS2* that could explain the skipping detected in P2. Exon 32 skipping in *AGL* was detected in P5 and was attributed to variant NC_000001.11(NM_000642.3):c.4260-1G>T previously identified by ES (data not shown). Both exons skipping result in out-of-frame transcripts. We also detected two aberrant isoforms of *ACADM* (HGNC:89) produced by the exonic variant NM_000016.5:c.542A>G previously misclassified as missense. One consists on a shortening of 58 bp of exon 7 (r.542_599del) producing an out-of-frame transcript (Fig. 1C), and the other consists on a 87 bp shortening, from which 58 bp correspond to exon 7 and 29 bp to exon 8 (r.542_628del), resulting in an in-frame deletion (Fig. 1C).

Allele-specific expression (ASE) was detected in three cases. Thus, the variants NM_000466.3:c.2097dup and NM_000019.4:c.841G>A present in *PEX1* (HGNC:8850) and *ACAT1*, respectively, displayed increased read numbers compared to their wild-type alleles (Fig. 1B), while aberrant *ACADM* transcripts caused by NM_000016.5:c.542A>G were increased (Fig. 1D). Besides, RT-qPCR quantification confirmed a significant reduction of 96% or 80% of *AGL* or *SLC2A1* mRNA expression in P5 and P4, respectively, when compared to at least three healthy controls (data not shown).

### Long-read sequencing

The observed transcriptional defects prompted us to search for non-coding variants by LRS targeted to cover up to 3 Mb of the genes of interest, thus encompassing the targeted *locus* and its regulatory landscape.

**Fig. 2:**
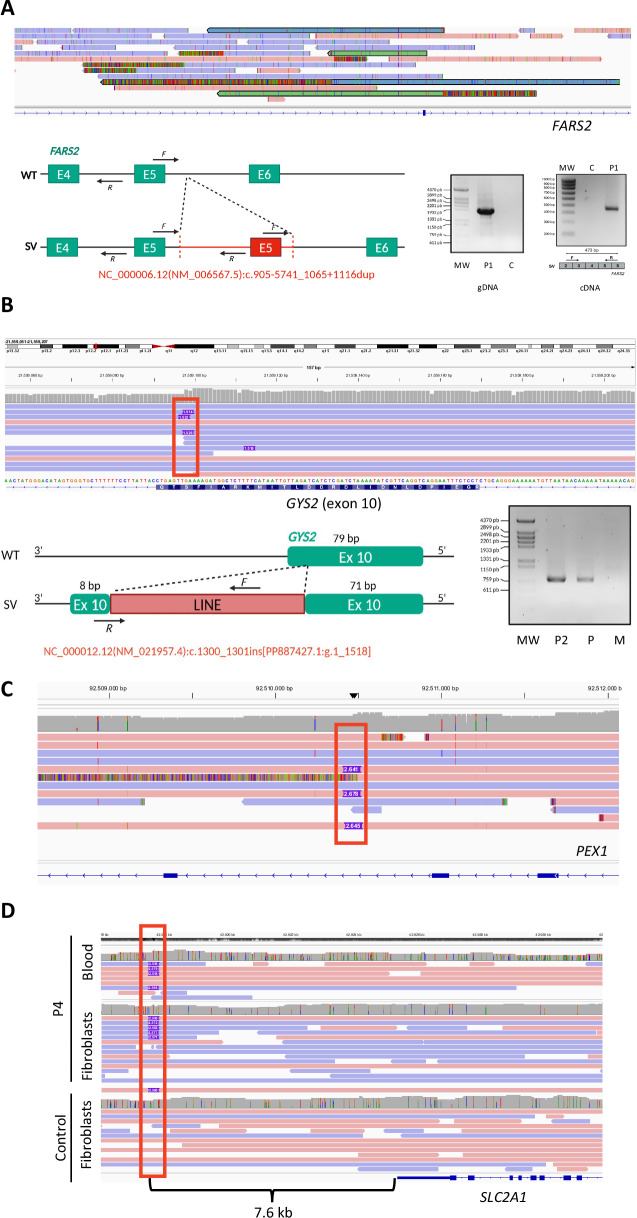
Long-read sequencing detects structural variants and insertions of transposable elements in participants 1, 2, 3, and 4 (P1, P2, P3, and P4). **A** Read visualization in the Integrative Genomics Viewer (IGV). The figure shows the pairs of linked reads in green and blue, both covering the duplication of exon 5. The left-lower part of the panel represents the specific PCR and RT-PCR amplification assay to confirm the exon 5 duplication in gDNA and cDNA. The primers used for RT-PCR are indicated in the figure. The upper part depicts the wild-type allele (WT) and the lower part the allele with the structural variant (SV), the duplication is marked in red. The forward (F) and reverse (R) primers used for the amplification are represented as black arrows. The right-lower part of the panel shows the agarose gel results of the specific PCR. **B** Read visualization in IGV. The figure shows, marked by a red rectangle, the insertion of a fragment of a Long Interspersed Nuclear Element (LINE) in the exon 10 of *GYS2*. The left-lower part of the panel represents the specific PCR amplification assay to confirm the LINE insertion. F and R primers are marked with black arrows. The right-lower part of the panel shows the agarose gel results. **C** Read visualization in IGV of the results for P3. The figure shows, marked by a red rectangle, the insertion detected in intron 8 of *PEX1*. **D** Read visualization in IGV of the results for P4. The figure shows, marked by a red rectangle, the LINE fragment insertion detected in the 3’ region of *SLC2A1*. MW molecular weight marker, C control sample, P parental sample, M Maternal sample.

In P1, we detected two reads showing a tandem duplication that includes the complete sequence of exon 5 of *FARS2* (Fig. 2A). This duplication was absent in DECIPHER. We designed a specific PCR and confirmed the disease-specific duplication (Fig. 2B, C), determining the variant’s breakpoints: NC_000006.12(NM_006567.5):c.905-5741_1065+1116dup that could not be previously mapped due to low coverage. Since we did not have parental samples available, we could not conduct segregation studies. Also, the low coverage of the LRS experiments did not allow for phasing the SV and the SNV identified in ES. The results suggest a duplication of exon 5 (161 bp) in the cDNA that likely causes a frameshift and ultimately leads to the degradation of the abnormal transcript (Fig. 2A). This additional genomic analysis suggests that the aberrant transcript observed in previous studies [28] is a result of this duplication.

We also identified TE insertions among three different participants. The sequence revealed two LINE1 fragments and one SVA in *GYS2*, *SLC2A1* and *PEX1*, respectively. In P2, a 1.5 kb fragment of a LINE_L1 (NM_021957.4:c.1300_1301ins[PP887427.1:g.1_1518]) was detected inserted in exon 10 of *GYS2* (Fig. 2B). This insertion likely causes the 79 bp skipping of exon 10 (previously observed) and the subsequent degradation of the aberrant mRNA due to the existence of a frameshift. The presence of the insertion was confirmed in the paternal allele by a specific PCR assay (Fig. 2B). Instead, the 2.6 kb SVA insertion of P3 occurred in intron 8 of *PEX1* (Fig. 2C). Finally, ONT sequencing of P4 identified a 2.5 kb LINE_L1 insertion 7.6 kb downstream of *SLC2A1* in both fibroblast and blood-extracted DNA (Fig. 2D). None of the three insertions has been reported in the control population before.

Regarding SNVs, we detected four novel variants. In P5, we found a novel deep intronic variant NC_000001.11(NM_000642.3):c.3259+927A>G in *AGL*, in *trans* with the previously detected variant. According to different in silico predictors, this variant increased the strength of a pre-existing splicing donor. For P6, a novel intronic variant was detected in *ACAT1* (NC_000011.10(NM_000019.4):c.941-60T>C) that eliminated a SRp55 binding site. Finally, for P7, we found two variants of uncertain significance with minor allele frequency below 1% and in *trans* with the exonic variant. One variant was found in the promoter region of *ACADM* NC_000001.11(NM_000016.5):c.-440T>C and the other was a deep intronic variant NC_000001.11(NM_000016.5):c.945+803A>C.

### Functional genomics reclassified three new variants as pathogenic

In addition to the RNA analysis, we assessed the effect of the SNVs detected in *AGL, ACAT1*, and *ACADM*, as well as the SV identified in *SLC2A1*, through functional genetic tests.

To analyze the intronic variant in *AGL*, a transcriptional profile analysis was conducted. The results suggest that the variant NC_000001.11(NM_000642.3):c.3259+927A>G results in a 105 bp PE insertion (Supplementary Fig. 1A) r.3259_3260ins[3259 + 818_3259 + 922] p.(Gly1087_Leu1532delinsAspPheHisLeuThrVal). Although this PE insertion is in frame, it generates a premature stop codon that presumably activates NMD.

A minigene analysis was done for the intronic variant detected in *ACAT1* in P6. The results suggest that the variant NC_000011.10(NM_000019.4):c.941-60T>C leads to the skipping of the 65 bp of exon 10 of *ACAT1* (Supplementary Fig. 1B).

For the identified *ACADM* promoter variant (NC_000001.11(NM_000016.5):c.-440T>C), the luciferase reporter assay showed slightly reduced transcriptional activity of this allele (data not shown). However, these results do not fully justify the reduced expression observed in the transcriptional profile (Fig. 1D).

**Fig. 3:**
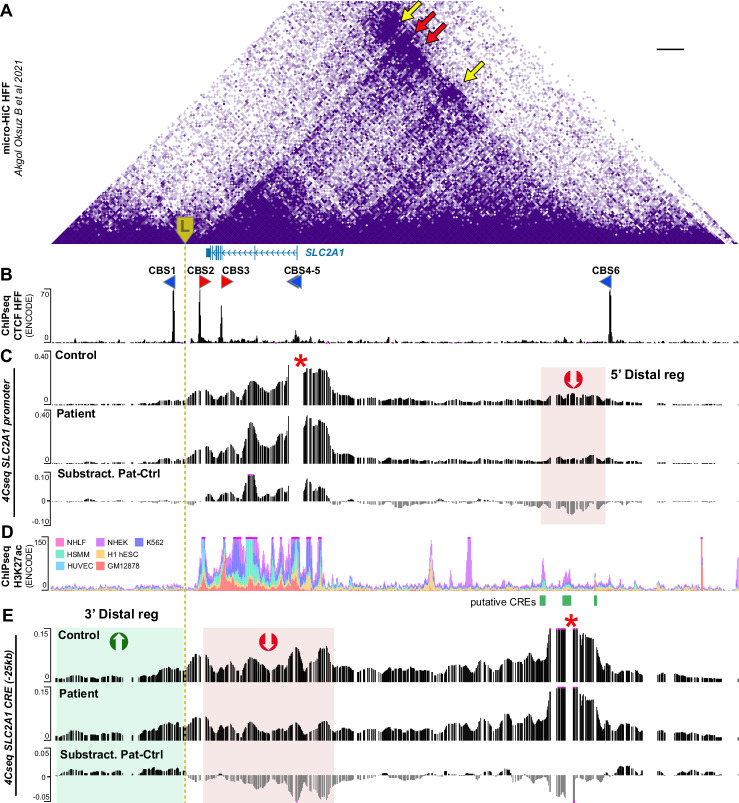
3D organization of the *SLC2A1* genomic region and conformational changes in participant 4 (P4). **A**,**B** Micro-HiC heatmap in HFFc6 (**A**) and chromatin immunoprecipitation coupled to sequencing (ChIPseq) profile for CTCF in NHLF cells from ENCODE (**B**). CTCF binding sites (CBS) with forward (red arrows) or reverse orientation (blue arrows) are displayed below the heatmap. The CBS orientation was predicted using the CTCFBS prediction tool [46]*. The SLC2A1* exonic structure is represented by blue boxes and arrows in the intronic regions indicate transcription orientation. The LINE insertion detected in P4 (L) is marked by a yellow box and dashed line. **C**,**E** Mean 4Cseq normalized profiles of *SLC2A1* promoter (**C**, upper graphs) or the *SLC2A1*-CRE (*cis* regulatory elements) cluster (**E**, upper graphs) viewpoints obtained from control and P4 fibroblasts. Each VP and the corresponding excluded region are marked by a red asterisk. The profiles include the average of two technical replicates for each sample and two biological replicates. The lower graph depicts the difference in contact score between the P4 sample and the mean of the controls. **D** ENCODE ChIPseq profiles for the H3K27ac epigenetic mark of different cell lines. The CRE constituting the cluster in the *SLC2A1* 5’ distal region are depicted by green rectangles.

To investigate the potential effect of the LINE insertion detected 7.6 kb into the *SLC2A1* 3’ region, we first investigated the 3D structure of this *locus*, exploiting available micro-HiC data [29, 30]. The *SLC2A1* region is organized in a TAD delimited by a single CTCF binding site (CBS) in a reverse orientation in its 3’ side, which interacts with two forward-oriented CBS of the 5’ TAD boundary (Fig. 3A, B; red arrows), in agreement with the loop extrusion model of TAD establishment [31]. The CBS of the 3’ TAD border also contacts the forward-oriented CBS near the *SLC2A1* TSS and in 5’ of the TAD (Fig. 3A, B; yellow arrows). Analysis of *SLC2A1* promoter interactions by 4Cseq in healthy individuals’ fibroblasts (controls), confirmed that *SLC2A1* contacts are largely restrained within its TAD, with the largest fraction of interactions spanning the *locus* and ~21 kb upstream of the gene TSS (Fig. 3C, Supplementary Fig. 2A, B). Besides, the *SLC2A1* promoter also strongly contacts a region 80 kb upstream (hereafter referred to as 5’ distal region) and near the CBS of the TAD border. According to ENCODE epigenetic profiles [32], this region contains several sequences enriched in the H3K27 acetylation mark (Fig. 3C; Supplementary Fig. 2A, B), a modification associated with active CRE [33].

In P4 fibroblasts, the *SLC2A1* promoter contacts with its 5’ distal region were strongly reduced compared to controls (Fig. 3C; Supplementary Fig. 2A, B, D). Conversely, proximal *locus* interactions tended to increase (although not statistically significantly), suggesting a more closed chromatin conformation. To confirm these results, we used as viewpoint (VP) the cluster of CRE located in the 5’ distal region (hereafter referred to as *SLC2A1*-CRE). This region strongly contacts the *SLC2A1* promoter and gene *locus* in controls (Fig. 3E; Supplementary Fig. 2C, E), while its interactions were significantly reduced in those of P4 (Supplementary Fig. 2C, E). Interestingly, the *SLC2A1*-CRE VP increased its interactions with the region immediately 5’ of the LINE insertion (Fig. 3E; Supplementary Fig. 2C, E). Of note, this tendency was observed in the comparison of P4 sample with that of either control, but it reached statistical significance only against control 1 or in P4 vs control averaged comparisons (Supplementary Fig. 2E), likely due to the variability in 4Cseq experiments. Instead, an equivalent region located on the opposite side of the *SLC2A1*-CRE VP (Supplementary Fig. 2E) did not show any clear tendency among conditions, supporting the specificity of the observed differences.

**Fig. 4:**
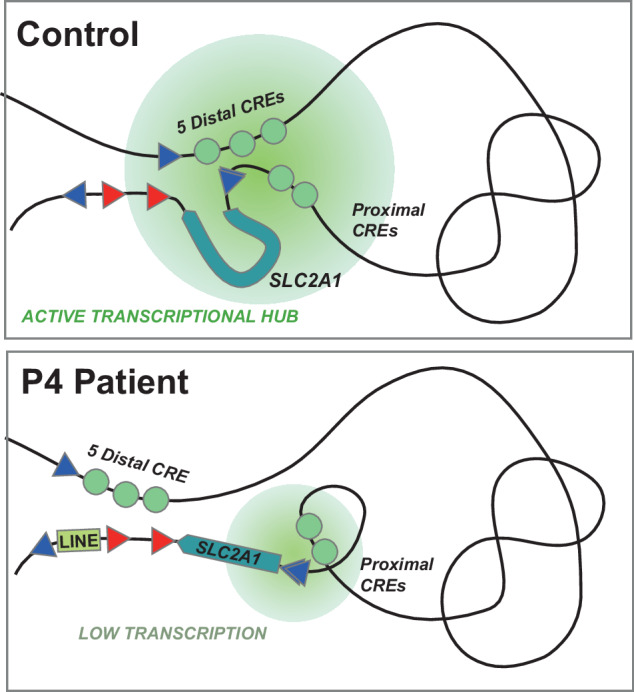
Scheme summarizing the chromatin conformation changes observed in control vs participant 4 (P4) fibroblasts. In control fibroblasts, the *SLC2A1* promoter contacts a *cis-regulatory* element (CRE) cluster located 80 kb upstream, likely via the looping of the CTCF binding site (CBS) of the 3’ topologically associating domain (TAD) border with the CBS of near the gene transcription start site (TSS), as well as those at the 5’ TAD border. This may result in the establishment of an active conformational hub (green shadowed circle) involving the *SLC2A1* locus (light blue box) and promoter, as well as proximal and distal enhancers (green circles; for simplicity, CRE located within the *SLC2A1* locus itself are not depicted), promoting or sustaining high *SLC2A1* transcription. Instead, in P4, the CRE cluster contacts are displaced towards the vicinity of the LINE insertion at the TAD border, possibly by CTCF-mediated looping between the CBS near the CRE cluster and those of the LINE. This leads to a reduction of CRE cluster-*SLC2A1* promoter contacts, and possibly, to an overall alteration of the structure of the conformational hub, weakening *SLC2A1* transcription.

Thus, our data suggest that the region located 80 kb upstream of the *SLC2A1* TSS contains a CRE cluster likely regulating *SLC2A1* expression and that the LINE insertion correlates with a decrease in the interactions between *SLC2A1* and this CRE cluster via rewiring of the contacts of the latter towards the vicinity of the LINE (Fig. 4).

## Discussion

Advances in sequencing technologies have led to the genetic diagnosis of many persons suspected to have a genetic disease. Nevertheless, diagnostic yield remains lower than expected [34]. Combining newer tools like LRS with multi-omics and functional analyses may help resolve more cases and shorten the diagnostic odyssey in IMD.

In this study, we show that applying adaptive sampling with ONT LRS makes it possible to phase and identify clinically relevant variants [35]. This approach is highly versatile, as it can target any genomic region without requiring prior assay design. As has been described by focusing LRS on specific genes, the method reduces both experimental and analytical barriers, ultimately facilitating its clinical adoption and contributing to a more comprehensive view of disease-associated variation [3].

In IMD, the presence of a biochemical biomarker eases the focus of genetic technologies to a limited number of genes. IMDs are identified in the neonatal screening program or after clinical suspicion and subsequent analysis by biochemical genetics. Nevertheless, molecular-genetic confirmation is needed. In our cohort of unsolved individuals, LRS has revealed the missing hit in six of them. Our results confirm that targeted LRS may be an adequate “next step” after genetic testing in the clinical setting when a candidate *locus* of interest is known. This technique has increased sensitivity to detect SV over srGS [35].

One of the major problems to conclude a definite genetic diagnosis is the assessment of the clinical relevance of VUS. This is more evident when an SV is detected due to the lack of public databases of population frequencies. Thus, different orthogonal analyses should be applied to validate these variants. RNA-Seq is one of the most powerful systems to evaluate variants affecting gene expression or splicing [36] if the gene is expressed in accessible tissues. Indeed, using the results of the RNA-Seq, we were able to identify defects in transcription (low expression levels, splicing defects, or ASE) that guided an LRS approach and, in the end, validated the clinical effect of the variants. The transcriptional defects have been related to intronic and promoter SNVs, duplications, or TE insertions.

In our knowledge, this is the first time the insertion of a TE has been reported to cause an IMD. Two of them were found inside the gene, one in exon 10 of *GYS2*, and the other was present in intron 8 of *PEX1*. This type of movement has been associated with pathology by inserting pseudoexons, leading to degradation via NMD [37]. The third TE insertion was detected 7.6 kb downstream of *SLC2A1* and could explain the reduction of the gene expression [38]. This result demonstrates that the movement of TE is a more common cause of disease than initially thought and that it should be implemented in the clinical setting in the future.

Finally, the use of LRS allows the detection of SNVs, indicating the potential use of this technology to identify all types of variants. We have identified SNVs affecting the splicing process in *AGL* and *ACAT1*. For the case of P6, the re-analysis of ES data allowed to detect the intronic variant in *ACAT1* in the visualization of BAM files, although it was not correctly called in the variant calling files (VCF). The filtering strategy to obtain the VFC limited variant detection to exons ±10 bp, therefore, leaving NC_000011.10(NM_000019.4):c.941-60T>C undetected. Thus, in autosomal recessive disorders, when a previously pathogenic variant is identified in a gene associated with the phenotype of a participant, all ES data should be carefully re-evaluated to ensure no disease-causing changes are being missed.

Predicting the effect of novel SVs is a complicated mission, especially when they fall outside the coding regions of genes [39]. Our 4Cseq experiments demonstrate that, in P4 fibroblasts, the *SLC2A1* promoter shows a significant decrease in contacts with a cluster of sequences enriched in enhancer epigenetic marks. This suggests that this region contributes to *SLC2A1* regulation and may explain the decrease in *SLC2A1* expression in P4. Although the magnitude of these alterations is relatively modest, this may be due to two factors. First, the patient is heterozygous for the LINE insertion, so the interactions of the other allele likely attenuate the observed differences. Second, the *SLC2A1*-CRE cluster may be active in a cell-type-specific manner, as suggested by ENCODE H3K27ac profiles, which show particularly low activity in fibroblasts (NHLF cells). While gene-enhancer contacts can occur across cell types, their strength often depends on enhancer activity [40]. Thus, experimenting with a cell type where the CRE cluster is active would likely reveal stronger effects. This also suggests that, although disruption of TAD internal organization may account for the transcriptional effects observed in patient-derived fibroblasts, the pathological phenotype of P4 may result from context-specific *SLC2A1* downregulation.

TE can shape gene regulatory landscapes through different mechanisms [41], including serving as CRE, or altering chromatin organization by bearing CBS [42]. Our 4Cseq data show that the *SLC2A1* promoter does not significantly change its interactions with the region near the LINE insertion in P4, unlike observed with the *SLC2A1*-CRE VP. This suggests that the LINE is unlikely to directly regulate *SLC2A1*. Instead, we identified putative CBS within the LINE sequence (Supplementary Fig. 3), although with some variation across LRS results. Despite confirming CTCF binding at the LINE is technically challenging, the presence of CBS near both the CRE cluster and the LINE may explain the observed contact rewiring. Nonetheless, other mechanisms may account for this phenomenon, such as the enhancer RNA- or TE-derived upstream antisense RNA–mediated looping described for Alu elements [43].

Finally, our results expand previous studies emphasizing the role of chromatin organization and gene-enhancer interactions in disease [7], and highlight the value of chromatin conformation capture methods for assessing the functional impact of novel genetic variants.

In the evolving landscape of precision and preventive medicine, several neonatal genomic sequencing pilot projects are exploring the potential use of ES or srGS to expand the number of detectable conditions beyond those identified by mass spectrometry and to enable early treatment before symptom onset [44]. Because these pilot genomic sequencing efforts mainly rely on short-read technologies, some of the variant types identified in our study would not be assessed using these approaches. Importantly, this does not affect the performance of established biochemical newborn screening, and no children currently identified through biochemical screening would be missed. However, our results show that some pathogenic variants associated with disorders not currently detected by biochemical newborn screening are challenging to capture with short-read ES or srGS, whereas they can be resolved using long-read sequencing. Our findings highlight the value of srGS and LRS for detecting pathogenic variants in non-coding regions and non-standard variant types [45] and point to future needs such as pangenome references and population-specific databases to fully enable these technologies in clinical and eventually newborn contexts.

In conclusion, this study has narrowed the diagnostic gap in IMD by integrating multiple omics data. We have expanded the mutational spectrum, identifying non-standard disease-causing variants. This enhanced knowledge will contribute to improving the sensitivity and specificity of genetic diagnosis for IMDs.

## Supplementary information

Supplemental material

## Data Availability

All the new variants have been submitted to the ClinVar database (https://www.ncbi.nlm.nih.gov/clinvar/) with the following accession numbers: SUB15762227, SUB15762348, SUB15762360, SUB15762410, SUB15762443, SUB15762466, SUB15762472 and SUB15768776. The data supporting the results of this study are available in the article and Supplementary Information or can be made available by contacting the corresponding author upon reasonable request.
