# Supplementary material for: Expanding the genetic landscape of inherited metabolic diseases using long-read sequencing and transcriptomic profiling
Source: Eur J Hum Genet. 2026 Jan 26;34(4):543–53. doi: 10.1038/s41431-025-01995-7 (PMC13046829; doi:10.1038/s41431-025-01995-7)
Supplement: Supplementary file 1 — Supplemental material [file 41431_2025_1995_MOESM1_ESM.pdf]

SUPPLEMENTAL FIGURE LEGENDS:

Supplemental Figure 1. Functional genomic studies performed for single nucleotide variants (SNVs) detected in participants (P) 5 and 6. A) Gel electrophoresis of the transcriptional study performed in fibroblast extracted RNA for participant P5 and a healthy control (C). Region containing exons 24 and 25 of *AGL* was amplified. **B)** Minigene design (upper part) and result of the transcriptional study after minigene transfection (lower part) containing the variant c.941-60T>C identified in P6 in *ACAT1*. V: vector.

Supplemental figure 2. Comparative analysis and quantification of 4Cseq profiles among samples. A) *SLC2A1* locus, CBS and CRE cluster annotations depicted as in Figure 3A. **B,C)** 4Cseq profiles and substraction plots of the *SLC2A1* promoter (**B**) or *SLC2A1*-CRE cluster (**C**) viewpoints (VPs). Each profile represents the average of two technical replicates per condition. The two control samples were averaged for the plots in Figure 3. The VPs and respective excluded regions are marked by a red asterisk. **D, E)** Plots showing the quantification of the *SLC2A1* promoter (**D**) or *SLC2A1*-CRE cluster (**E**) 4Cseq signal in control and P4 samples along the different regions analyzed in this study. Violin plots show the comparison of the averaged technical and biological replicates of controls vs P4 conditions. Box and whiskers plot represent the statistics of each technical and biological replicate separately. The vertical boxes represent the 25th and 75th percentiles of contact intensity distribution within the selected region the median depicted by a horizontal line. The mean of the distribution is depicted with a "+" symbol. The whiskers represent the Q1 − 1.5 × IQR and the Q3 + 1.5 × IQR sides of the

distribution. Statistical significance of violin plot and box and whickers data were calculated using a two tailed unpaired T-test, or a two tailed nested T-test, respectively.

Supplemental figure 3. Analysis of CBS in the *SLC2A1* genomic region and LINE insertion. A) Micro-HiC heatmap and *SLC2A1* locus, CBS and CRE cluster annotations depicted as in Figure 3. The PWM matrices for which the CTCFBS predictor tool identified the putative CBS and the score and direction of the predicted site are depicted on each side of the heatmap plot. The LINE insertion (L) is depicted by a yellow box and dashed line and represented by a blue box, with the point indicating the orientation of the inserted element, at the bottom of the panel. Putative CBS (pCBS) identified by the CTCFBS predictor tool are represented with blue and red triangles as the CBS of the *SLC2A1* genomic region. **B)** Nucleotide alignment of the LINE sequence obtained from LRS experiments performed on blood or fibroblast-extracted DNA. The reference weight matrix and CTCBS predictor tool hits score for each of the putative sites are provided on the right.

A

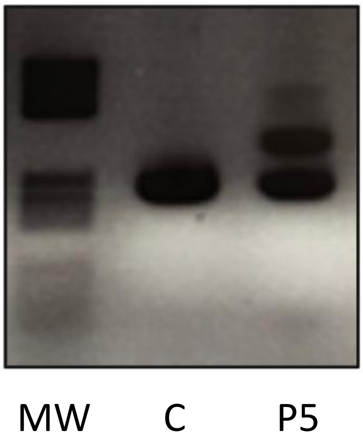
MW
C
P5

B

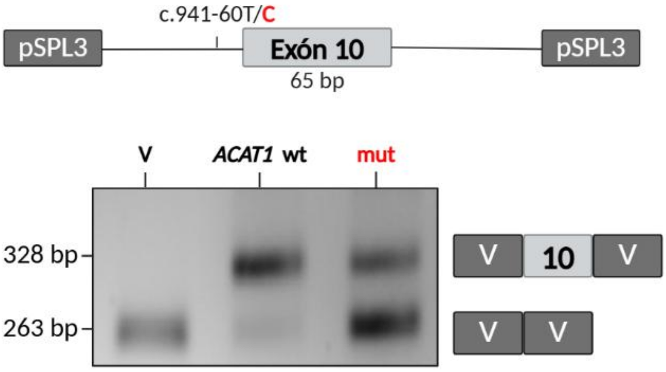
c.941-60T/C
pSPL3
Exón 10
65 bp
pSPL3
V
ACAT1 wt
mut
328 bp
263 bp
V
10
V
V
V

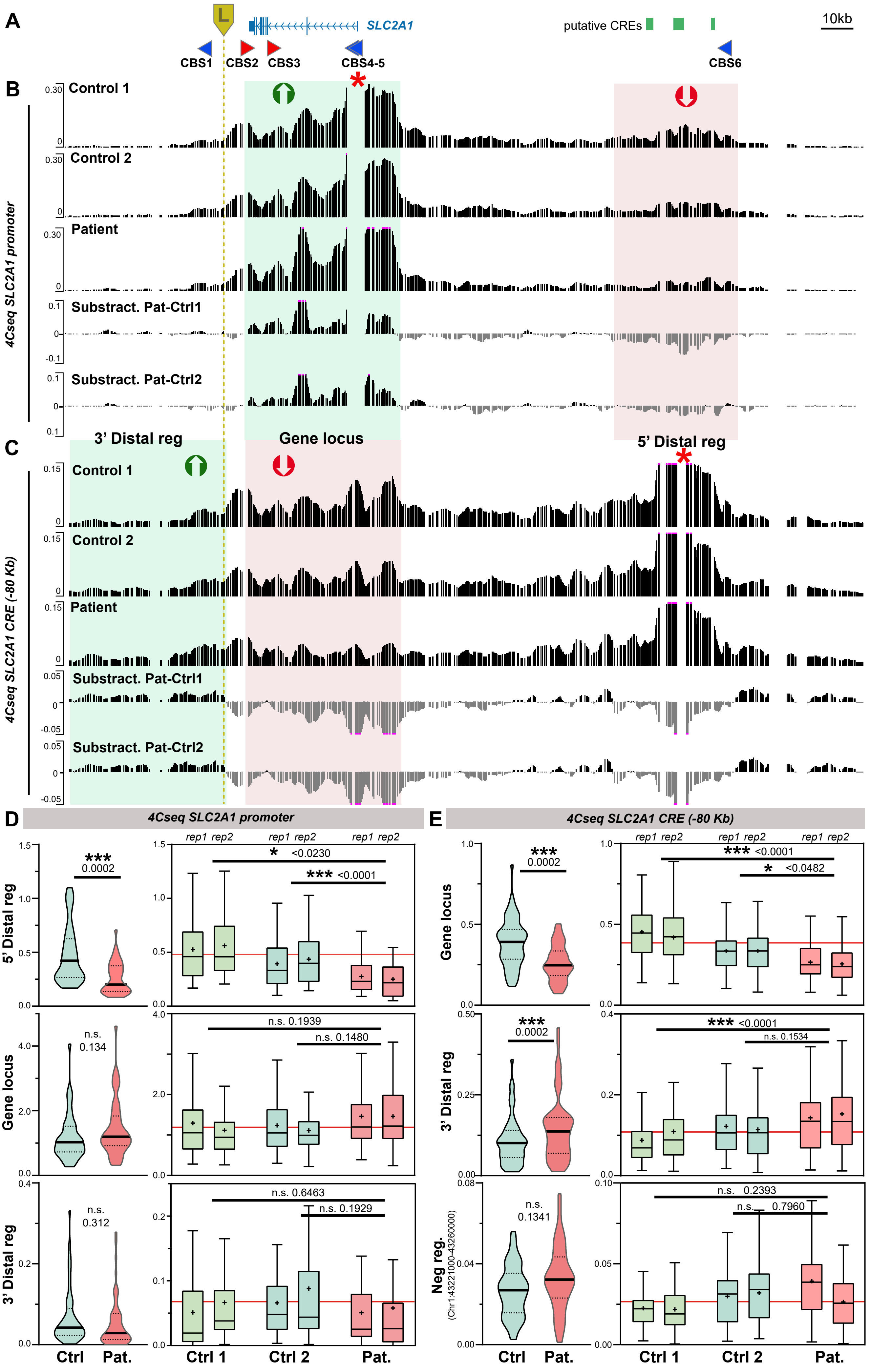

A
SLC2A1
putative CREs
10kb
CBS1
CBS2
CBS3
CBS4-5
CBS6
B
4Cseq SLC2A1 promoter
Control 1
Control 2
Patient
Substract. Pat-Ctrl1
Substract. Pat-Ctrl2
C
4Cseq SLC2A1 CRE (-80 Kb)
3' Distal reg
Gene locus
5' Distal reg
Control 1
Control 2
Patient
Substract. Pat-Ctrl1
Substract. Pat-Ctrl2
D
4Cseq SLC2A1 promoter
5' Distal reg
Gene locus
3' Distal reg
*** 0.0002
n.s. 0.134
n.s. 0.312
rep1 rep2
* <0.0230
*** <0.0001
n.s. 0.1939
n.s. 0.1480
n.s. 0.6463
n.s. 0.1929
Ctrl
Pat.
Ctrl 1
Ctrl 2
Pat.
E
4Cseq SLC2A1 CRE (-80 Kb)
Gene locus
3' Distal reg
Neg reg.
(Chr1:43221000-43260000)
*** 0.0002
*** 0.0002
n.s. 0.1341
rep1 rep2
*** <0.0001
* <0.0482
*** <0.0001
n.s. 0.1534
n.s. 0.2393
n.s. 0.7960
Ctrl
Pat.
Ctrl 1
Ctrl 2
Pat.

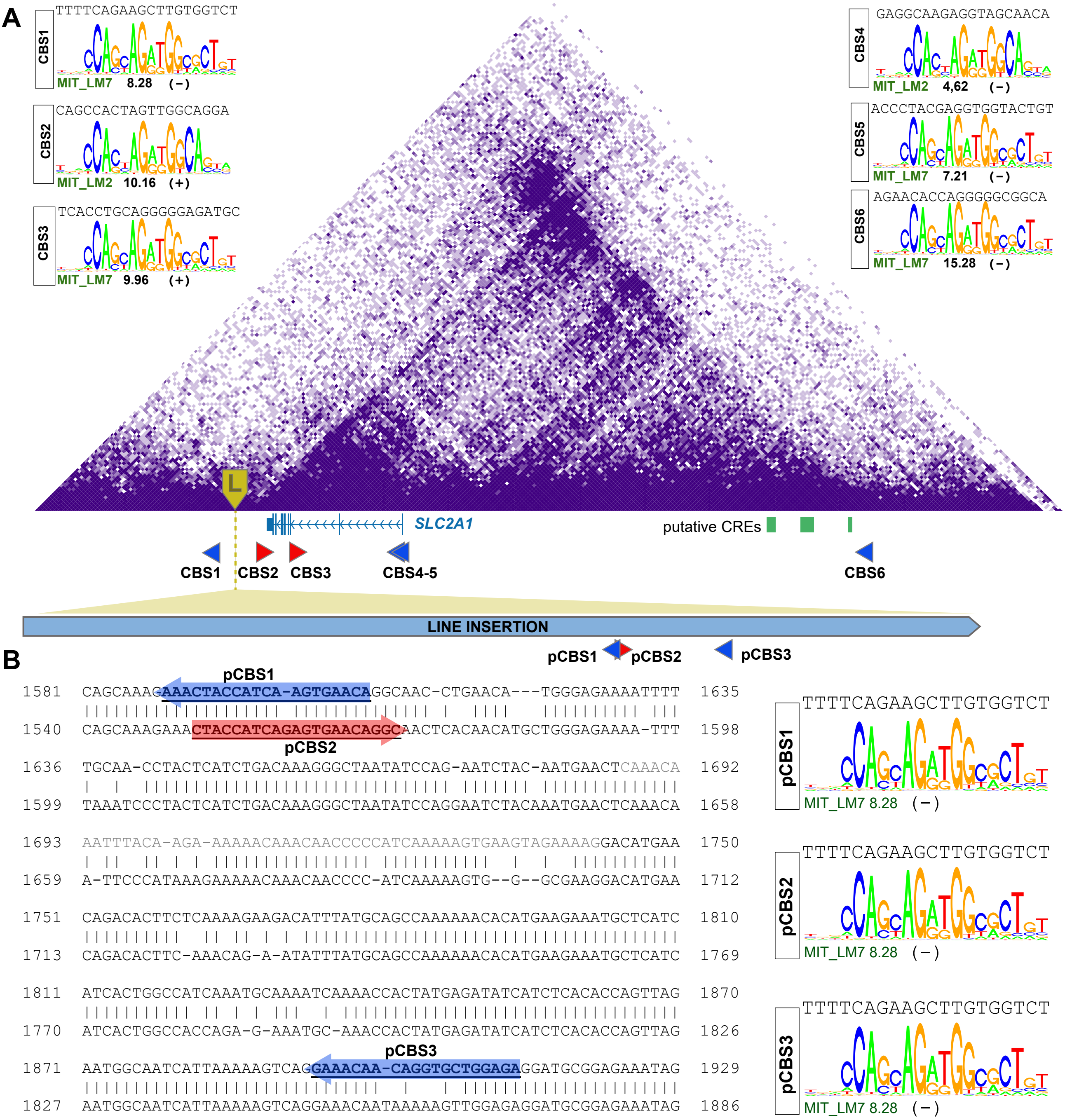
A
CBS1
TTTTCAGAAGCTTGTGGTCT
MIT_LM7 8.28 (−)
CBS2
CAGCCACTAGTTGGCAGGA
MIT_LM2 10.16 (+)
CBS3
TCACCTGCAGGGGGAGATGC
MIT_LM7 9.96 (+)
CBS4
GAGGCAAGAGGTAGCAACA
MIT_LM2 4,62 (−)
CBS5
ACCCTACGAGGTGGTACTGT
MIT_LM7 7.21 (−)
CBS6
AGAACACCAGGGGGCGGCA
MIT_LM7 15.28 (−)
L
SLC2A1
putative CREs
CBS1 CBS2 CBS3 CBS4-5 CBS6
LINE INSERTION
pCBS1 pCBS2 pCBS3
B
pCBS1
1581 CAGCAAAGAAACTACCATCA-AGTGAACAGGCAAC-CTGAACA---TGGGAGAAAATTTT 1635
1540 CAGCAAAGAAACTACCATCAGAGTGAACAGGCAACTCACAACATGCTGGGAGAAAA-TTT 1598
pCBS2
1636 TGCAA-CCTACTCATCTGACAAAGGGCTAATATCCAG-AATCTAC-AATGAACTCAAACA 1692
1599 TAAATCCCTACTCATCTGACAAAGGGCTAATATCCAGGAATCTACAAATGAACTCAAACA 1658
1693 AATTTACA-AGA-AAAAACAAACAACCCCCATCAAAAAGTGAAGTAGAAAAGGACATGAA 1750
1659 A-TTCCCATAAAGAAAAACAAACAACCCCC-ATCAAAAAGTG--G--GCGAAGGACATGAA 1712
1751 CAGACACTTCTCAAAAGAAGACATTTATGCAGCCAAAAAACACATGAAGAAATGCTCATC 1810
1713 CAGACACTTC-AAACAG-A-ATATTTATGCAGCCAAAAAACACATGAAGAAATGCTCATC 1769
1811 ATCACTGGCCATCAAATGCAAAATCAAAACCACTATGAGATATCATCTCACACCAGTTAG 1870
1770 ATCACTGGCCACCAGA-G-AAATGC-AAACCACTATGAGATATCATCTCACACCAGTTAG 1826
pCBS3
1871 AATGGCAATCATTAAAAAGTCAGGAAACAA-CAGGTGCTGGAGAGGATGCGGAGAAATAG 1929
1827 AATGGCAATCATTAAAAAGTCAGGAAACAATAAAAAGTTGGAGAGGATGCGGAGAAATAG 1886
pCBS1
TTTTCAGAAGCTTGTGGTCT
MIT_LM7 8.28 (−)
pCBS2
TTTTCAGAAGCTTGTGGTCT
MIT_LM7 8.28 (−)
pCBS3
TTTTCAGAAGCTTGTGGTCT
MIT_LM7 8.28 (−)

Supplemental Table 1. Genomic regions used in adaptive sampling for long-read sequencing

Participant	Gene	Region
P1	*FARS2*	GRCh38, chr6:4032365-7032364
P2	*GYS2*	GRCh38, chr12:20029135-23029134:
P3	*SLC2A1*	GRCh38, chr1:41512390-44531184
P4	*PEX1*	GRCh38, chr7:91015143-94015143
P5	*AGL*	GRCh38, chr1:98537840-101527009
P6	*ACAT1*	GRCh38, chr11:106727193-109688945
P7	*ACADM*	GRCh38, chr1:74242704-77242704

Supplemental Table 2. Oligonucleotides used for Circular Chromatin Conformation Capture experiments.

Oligonucleotide name	Sequence 5'-3'
Promoter Reading	TACACGACGCTCTTCCGATCTNNNNNAAGTGTTTAGAACAGCGTCT
Promoter Amplification	ACTGGAGTTCAGACGTGTGCTCTTCCGATCTAGAGAGAACGAGCCGATC
CRE Reading	TACACGACGCTCTTCCGATCTNNNNNAGAAGGGCTTTTGGAGAATGTG
CRE Amplification	ACTGGAGTTCAGACGTGTGCTCTTCCGATCTACCAGTAACTGACTCTGTATGTC
Library preparation F	AATGATACGGCGACCACCGAGATCTACACTCTTTCCCTACACGACGCTCTTCCGATCT
Library preparation R	CAAGCAGAAGACGGCATACGAGATXXXXXXGTGACTGGAGTTCAGACGTGTGCT

5' overhang regions (hybridization sequence for Illumina Truseq) are marked in blue, underlined regions specify adapters P5 (forward primer) and P7 (reverse primer). XXXXXX indicate regions where Truseq Indexes were added to identify each sample.
